# Cesarean Section or Natural Childbirth? Cesarean Birth May Damage Your Health

**DOI:** 10.3389/fpsyg.2019.00351

**Published:** 2019-02-21

**Authors:** Hongyan Chen, Dingliang Tan

**Affiliations:** ^1^School of Psychology, Nanjing Normal University, Nanjing, China; ^2^School of Psychology, Xinxiang Medical University, Xinxiang, China; ^3^School of Education Science, Nanjing Normal University, Nanjing, China

**Keywords:** Cesarean section, children, natural childbirth, psychological aspects, neural mechanisms

## Abstract

The increasing popularity of Cesarean birth has become a social concern in many countries. This paper reviews the literature on the effects of Cesarean section on children’s psychological health. The results show that Cesarean birth may have adverse effects on children’s sensory perception, sensory integration ability, neuropsychiatric development, and the infant-mother relationship. However, there remain deficiencies in extant research methods, research content, subject groupings, and interpretation of research results. Future research should improve research methods, broaden the research content, and refine the grouping of children born by Cesarean section. The exploration of neural mechanisms is also needed, as well as research directed toward suggesting effective interventions to reduce unnecessary Cesarean sections.

## Introduction

Human reproduction guarantees the continuation and evolution of the human species; thus, births are significant events, which many view as sacred. In general, human birth can be divided into four categories: natural delivery, assisted delivery, Cesarean section due to medical factors, and Cesarean section due to social factors. It is well known that Cesarean section has an irreplaceable role in the rapid resolution of parturition under certain medical conditions, such as dystocia, intrauterine distress, fetal position, and so on. Therefore, Cesarean section due to medical indications is a necessary operation. However, in the last two decades, Cesarean section due to social factors has become an increasingly popular choice (Muula, 2007; Bu, 2008; Khadem and Khadivzadeh, 2009; Khalaf et al., 2015; Curran et al., 2016).

Cesarean section was originally a surgical solution to solve the problems associated with difficult labor, but now there are no controls over its use. This increasing popularity has led to a rapid growth in the number of Cesarean section operations worldwide. The percentage of births delivered by Cesarean section has increased in the United Kingdom from 18% in 1997 to 25% in 2010, and in the United States, the percentage has increased from 27% in 1997 to 31.8% in 2011 (Curran et al., 2016). A survey by the World Health Organization showed that the average percentage of deliveries by Cesarean section in developed countries has reached 25%, which is considerably greater than the 15% recommended by the World Health Organization (Curran et al., 2016). In Asia, the ratio is even higher. In Iran, the proportion of Cesarean section operations is close to 40%, and in some areas the proportion is as high as 52.8% (Khadem and Khadivzadeh, 2009). In China, the percentage has reached 34.9% (Tian, 2017), however, in some rural areas, this proportion is even higher.

Natural childbirth is the inevitable physiological process of human reproduction and it has many positive effects. For example, in spontaneous labor, the first contact between mother and child is timely, which is very important for establishing mother-child coordination and the child’s psychological development (Huang et al., 2004). However, Cesarean section is an unnatural mode of delivery. After Cesarean section, first contact time is delayed due to anesthesia, pain of surgical incision, emotional tension, and other factors, which affects the psychological development of newborns. In recent years, researchers have investigated the impact of Cesarean section on children’s psychological health. This paper reviews the extant research to provide points of reference for standardizing the occupational behaviors of obstetricians and promoting the physical and mental health of children.

## Criteria for Inclusion of Studies in This Review

In this review, we focus on the effects of Cesarean section on children’s psychological health, such as sensory perception, sensory integration ability, neuropsychiatric development, and infant-mother relationship. We present a selective review of articles addressing the effects of Cesarean section on children’s psychological health. Thus, studies on the effects of Cesarean section on children’s physiological aspects were excluded, for example, on children’s obesity, asthma, allergy, autoimmune disorder, gastro-intestinal disorders, and so on.

The primary criterion for inclusion in this review was that publications were original research published in a peer-reviewed scientific journal. Medline, PubMed, EBSCO, and Psychlit were used for article searches. Search terms were as follows: “Cesarean section,” “natural childbirth,” and “children.” However, since our focus was on the effects of Cesarean section on children, comparisons with natural and assisted delivery groups were excluded. Additionally, as we focused on the effects of Cesarean section on children’s psychological health, studies of the effects of Cesarean section on the puerperas’ psychological health were excluded. The second criterion for inclusion was that the study must have a sample size of at least 10 subjects (per group).

## Effect of Cesarean Section on Children’s Psychological Health

### Effect of Cesarean Section on Children’s Sensory Perception

Sensory perception refers to the processing by the human brain of objective sensory inputs that have been transduced by the sensory organs. Such perception is the basis for all the advanced psychological processes, which are of great significance for individual development. Research has indicated that, compared with natural childbirth, Cesarean section has negative impacts on children’s senses of smell, touch, and visual ability.

Varendi et al. (2002) studied the impact of Cesarean section on infants’ olfactory performance among 31 individuals who received Cesarean section, 15 of whom underwent uterine contraction before Cesarean section and 16 of whom did not experience uterine contraction. The two groups of newborn babies were exposed to a certain odor for 30 min after birth. They were then exposed to familiar and novel smells on both sides of their faces 80 h later. The experimental materials used were two odorant-saturated gauzes. Infants’ responses to the odorants were videotaped. Neonates who had experienced contractions showed a preference for familiar scents, while newborns who did not experience contractions did not have this preference; that is, the latter could not recognize the familiar scent. This suggests that contractions may promote the newborn child’s olfactory learning ability. However, in this study, a non-Cesarean section group was not assessed. Therefore, there were limitations regarding the sampling.

Through two decades of clinical observations, Mao and Jing (2005) found that newborns delivered via Cesarean section did not like to be touched or hugged as compared with newborns delivered via natural childbirth. The neonates expressed stress regarding physical contact with their mothers. Furthermore, emergency Cesarean section operations had a greater impact on neonates’ sense of touch compared to those born by planned Cesarean section. Children born through emergency Cesarean section were prone to tactile resistance due to the experience of birth trauma.

Approximately 80% of sensory information that humans process is visual in nature, and visuospatial perception is of great importance for the development of children’s learning abilities. Huang et al. (2005) adopted the Benton Visual Retention Test to assess the visuospatial perception of children in the third and fourth grades of school who had been born by Cesarean section due to social factors or born by vaginal delivery. The two groups were balanced in terms of school, age, gender, family, and other factors. The results showed that these children were less able to reproduce figures compared to a group of children born by vaginal delivery; that is, the visual memory and visuospatial perception abilities of the former group were poorer than those of the latter group. Additionally, among the various types of errors made on the Benton Visual Retention Test, the average number of errors made by the Cesarean section group was significantly higher than that of the control group. This indicates that the visuospatial perception ability of children in the Cesarean section group was poorer than that of the control group.

### Effect of Cesarean Section on Children’s Sensory Integration

Sensory integration refers to the ability of individuals to utilize sensory information from different parts of the body and to respond to these sensory inputs appropriately. Sensory integration plays a crucial role in children’s learning ability and social adaptability. Studies have found that the sensory integration ability of children born by Cesarean section is worse than that of children born by natural childbirth (Bu et al., 2008; Kong et al., 2009; Tian, 2009; Yuan et al., 2009). Tian (2009) also found that children born by Cesarean section due to medical factors and Cesarean section due to medical factors both showed poorer sensory integration ability than children born by natural childbirth.

Overall, the results indicate that Cesarean section has negative impacts on children’s senses of smell, touch, and vision and on sensory integration abilities. However, few studies have considered the effects of Cesarean section on children’s perceptual abilities; research on this topic needs to be further strengthened.

### Cesarean Section and Neuropsychiatric Disorders in Children

#### Cesarean Section and Children’s Attention Deficit/Hyperactivity Disorder

Attention deficit/hyperactivity disorder (ADHD) is characterized by a persistent pattern of inattention and/or hyperactivity-impulsivity that interferes with functioning (American Psychiatric Association [APA], 2013). Children with ADHD may experience deficits in cognitive abilities, issues with social and adaptive functioning, as well as disturbances in motivation and emotion. Research has shown that Cesarean delivery may increase the risk of ADHD in children.

Song et al. (2008) found the proportion of children with ADHD in a natural delivery group, assisted delivery group, and Cesarean section group were 6.25, 4.76, and 11.6%, respectively, differences among groups were statistically significant. Additionally, a Swedish study assessed a population cohort of 722,548 newborns registered by the Swedish National Bureau of Statistics during 1990–2008 (Curran et al., 2016). It was found that the hazard ratio (HR) of the association between elective Cesarean section compared with natural delivery regarding ADHD was 1.15. The HR of the association between emergency Cesarean section and ADHD was 1.16. However, among siblings the association only remained for emergency Cesarean section. This indicates that the relationship between ADHD and Cesarean delivery mode may be related to medical indications that necessitate emergency Cesarean section. Therefore, the relationship between Cesarean delivery mode and ADHD needs to be further explored.

#### Cesarean Section and Autism Spectrum Disorder in Children

Autism spectrum disorder (ASD) is characterized by impaired social interactions and communication, with the presence of restricted interests and repetitive behaviors (American Psychiatric Association [APA], 2013). Studies have found that there may be a link between Cesarean section and ASD (Dodds et al., 2011; Yip et al., 2016).

A Canadian study found that children born by Cesarean section were 1.23 times more likely to experience ASD than children born by natural childbirth (Dodds et al., 2011). An epidemiological study in 2016 used a cohort design to investigate ASD among 5 million children in Norway, Sweden, Denmark, Finland, and Australia (Yip et al., 2016). Compared with vaginal delivery, the overall adjusted OR for ASD following Cesarean section was 1.26. Across the five countries, emergency or planned Cesarean section was consistently associated with a modestly increased risk of ASD from gestational weeks 36–42 when compared with vaginal delivery (Yip et al., 2016). However, Curran et al. (2015b) found that compared with children in a spontaneous delivery group, children born by Cesarean section were approximately 20% more likely to be diagnosed as having ASD. However, the association did not persist when using sibling controls, implying that this association may be due to familial confounding of genetic and/or environmental factors. Therefore, the relationship between Cesarean delivery and ASD needs to be further explored.

Overall, among the studies of the impact of Cesarean section on children’s neuropsychiatric development, population cohort designs have predominated. Such studies typically used the risk ratio as an indicator of the existence of relationships between Cesarean delivery patterns and ADHD and ASD. Recently, researchers have used sibling-control designs. There are differences in the results obtained by the two methods. Thus, the relationship between Cesarean section and children’s neuropsychiatric development needs to be further explored.

#### Cesarean Section and Schizophrenia in Children

Schizophrenia is a serious and disruptive mental disorder that has a substantial effect on public health (Collins et al., 2011). Schizophrenia and Cesarean birth are associated (Verdoux et al., 1997; Boksa and Ei-Khodor, 2003; Fond et al., 2016). For instance, Verdoux et al. (1997) found that the incidence of early onset schizophrenia in a group of Cesarean births was 10 times higher than the incidence of late onset schizophrenia, which suggests that Cesarean birth is associated with the early onset of schizophrenia. The natural childbirth group did not exhibit this characteristic.

### The Effect of Cesarean Section on the Mother-Infant Relationship

The emotional relationship between infant and mother is intense. A healthy maternal-infant relationship plays an important role in the successful socialization of children and the robust development of their personality. Cesarean delivery is not conducive to the establishment of a healthy relationship between mother and infant.

Numerous studies have found that mothers with children born by Cesarean section have far poorer mother-infant relationships than mothers who experienced spontaneous delivery (Green et al., 1991; Hillan, 1991; Simons et al., 1992). Studies have found that Cesarean section has a negative effect on establishing a safe pattern of parent-child attachment (Dimatteo et al., 1996; Lobel and Deluca, 2007; Herguner et al., 2012). Mothers with children born by Cesarean section have more negative evaluations of their children (Dimatteo et al., 1996). Mothers in a Cesarean section group had significantly lower scores on a mother-child attachment scale than mothers in a natural birth group (Herguner et al., 2012). Mothers in a natural birth group have also been shown to be more motivated to take care of newborns and felt less tired than mothers in a Cesarean section group, who were more likely to fail in their efforts to care for their newborns (Wiklund et al., 2009).

Questionnaires and clinical observations have been the most popular research tools for investigating the impact of Cesarean section on the mother-infant relationship. Studies have investigated the scores of puerpera on mother-child attachment scales after delivery, and observed the interaction between puerpera and newborns after delivery. The results indicated that Cesarean section has negative impacts on the mother-infant relationship.

## The Mechanism of Negative Impacts of Cesarean Birth on Child Development

### The Mechanism of Negative Impacts of Cesarean Birth on Children’s Sensory Perception and Sensory Integration

The newborn child’s olfactory learning ability may be promoted by contractions. The fetus experiences the mother’s contractions during natural delivery, whereas most children delivered via Cesarean section lack this experience. From this perspective, natural childbirth may help to promote the newborn child’s olfactory learning ability. Researchers have noted that labor contractions also stimulate noradrenergic neurons in the locus coeruleus and thereby increase brain arousal. This activation may account for the state of alertness that is typical of human neonates within the first 1–2 h of birth, as well as their heightened responsiveness to stimulus input (and possibly increased learning efficiency) within that brief time window (Svensson, 1987; Lagercrantz, 1996).

Regarding the child’s sense of touch, newborns delivered by Cesarean section do not experience compression within the birth canal. The first touch they receive is that of the operation-related medical staff. This is not the gentle touch required by newborns, and such inappropriate physical contact engenders pain in the neonate.

Concerning the child’s visuospatial ability, researchers have noted that unbalanced development of visuospatial ability in infants following Cesarean may be closely related to the absence of sensory learning associated with natural delivery. Similarly, the lack of tactile learning associated with Cesarean section may be one reason for sensory integration disorders in children (Guo et al., 2000; Wang, 2000). Cesarean section is an interventional delivery, during which neonates are delivered passively in a short period of time. As such, infants delivered by Cesarean section do not experience early tactile pressure and the associated learning; consequently, some such infants show no sense of proprioception and may develop other sensory integration disorders. Due to the extrusion of the birth canal, children born by natural childbirth experience cohesion, descent, flexion, internal rotation, and extension over a short time period; thus, they undergo marked tactile, proprioceptive, and vestibular learning.

In conclusion, a possible reason for the negative impact of Cesarean section on children’s sensory perception and sensory integration ability is the lack of sensory learning associated with natural delivery. However, the mechanisms underlying the adverse effects of Cesarean section on children’s sensation have not been fully explored; further study is needed.

### The Mechanisms of Negative Impact of Cesarean Birth on Children’s Neuropsychiatric Disorders

Concerning the relationship between Cesarean delivery mode and ADHD, animal experiments have been used to determine why Cesarean section increases the risk of ADHD in children. Juárez et al. (2010) considered 120 randomly-chosen newborn rat pups that had been delivered by vaginal birth (VAG), Cesarean section only (C-only), or Cesarean section accompanied by an absence of oxygen (C+Anoxia). Neurons were extracted from living pups from both sides of the medial prefrontal cortex (PFC), nucleus accumbens (NAcc), and hippocampal CA1 regions at different postnatal ages over time. Subsequently, the rats were sacrificed and brain PFC, NAcc, and hippocampal CA1 regions sliced and each section examined by microscopy. Dendritic tree length and density were compared at each postnatal age at which samples were collected and after the brain was removed. Cesarean section, regardless of anoxia, affected prepubertal development of PFC and hippocampal CA1 neurons, as well as the NAcc medium spiny neurons. The dopamine levels in the NAcc were increased in the rats born by Cesarean section, with or without anoxia. The results of this study indicate that neural changes can affect dopamine function in the PFC and NAcc, which may be associated with dopamine-related disorders such as schizophrenia, ADHD, and drug addiction.

Many neuroanatomical networks, including the prefrontal cortex and anterior basal ganglia, are rich in dopamine (Purper-Ouakil et al., 2005). Dopamine, which is very sensitive to perinatal factors, is involved in the regulation of attention and task-execution. Cesarean delivery can alter the amount of neurotransmitters and the mechanisms by which neurotransmitters are released after birth, thus increasing the risk of attention deficit disorder (Ei-khodor and Boksa, 2002).

Studies have sought evidence for a relationship between Cesarean delivery mode and ASD. The incidence of ASD following Cesarean births may primarily relate to two factors. First, anesthesia during childbirth is an important factor. Taiwanese studies have assessed the effect of general anesthesia in Cesarean births and local anesthesia on autism in children (Chien et al., 2015). Children who underwent general anesthesia for Cesarean section had a higher risk of autism compared to a control group. However, the incidence of autism in the Cesarean group who underwent local anesthesia was not significantly different from that in the spontaneous labor group. Additionally, the researchers also noted that, compared with the natural birth group, girls in the Cesarean group who had experienced general anesthesia were twice as likely as boys to experience autism, suggesting that girls may be more sensitive to the long-term effects of general anesthesia than boys. This also highlights that neurotoxicity of general anesthetic agents affects children’s future neurological development. Rice and Barone (2000) found that damage to the brain in the early stages of infancy can affect the development of synapses in certain brain regions, and subsequently delay or influence the future development of those regions.

Second, postpartum anesthesia and surgical trauma delay the time of first contact between mother and child and of breastfeeding. The delayed parent-child interaction affects child-attachment, which can greatly harm the psychological development of children born by Cesarean section and cause behavioral problems in children (Di, 2009).

The influence of Cesarean section on schizophrenia may be the result of many factors. First, those born by Cesarean section are not exposed to the mother’s vaginal microbiota at birth; hence, their intestinal microbiota differ from that of natural births (Makino et al., 2013). The difference in microbiota continues into adolescence and early adulthood (Mueller et al., 2015). Microbiota play an important role in the development of the brain and in the occurrence of major neurological disorders (Collins et al., 2012); thus, the lack of microbiota caused by Cesarean section may play a role in the onset of schizophrenia. Second, dopamine receptors have been implicated in the pathogenesis of schizophrenia (El-khodor and Boksa, 2001; Boksa et al., 2002; Novak et al., 2011; Fond et al., 2015). Long-term changes in dopamine receptors caused by Cesarean section may also account for schizophrenia. Additionally, perinatal brain injuries caused by emergency Cesarean section, particularly intrauterine fetal hypoxia, also constitute an etiological risk factor for schizophrenia (Boksa and Ei-Khodor, 2003).

### The Mechanisms of the Negative Impact of Cesarean Section on the Mother-Infant Relationship

Functional magnetic resonance imaging studies have also investigated mother-infant attachment patterns (Swain et al., 2008). After 2 weeks of delivery, mothers in the spontaneous delivery group were more sensitive to their children’s cries and responded more positively compared to mothers in the Cesarean section group. In addition, the former group showed more activity in brain regions such as the frontal gyrus, middle fusiform gyrus, anterior superior lobe, caudate nucleus, thalamus, hypothalamus, amygdala, and pons. The study also showed that some infant behaviors activated the mother’s neuronal circuits related to mood, motivation, attention, and empathy. Among the key factors affecting the neuronal circuit of the mother’s brain, and ultimately the maternal-infant relationship, the mode of birth delivery plays an important role (Swain et al., 2007; Swain and Lorberbaum, 2008). In the course of spontaneous labor, the contraction of the uterus and the movement of the vagina and cervix stimulate the mother’s pulsed release of hormones from the posterior pituitary gland (Leckman and Herman, 2002). Animal experiments have shown that the posterior pituitary hormone is an important intermediary in maternal behavior (Kendrick et al., 1992; Morgan et al., 1992; Porter et al., 2002; Poindron, 2005). The mode of Cesarean delivery deprives the vagina and cervix of the movements involved in spontaneous labor, thus affecting the release of hormones from the pituitary gland. This will affect the response of the mother’s brain to infant behavior in the early postpartum period.

Overall, the mechanism underlying the negative impact of Cesarean section on child development needs to be further elucidated; indeed, the mechanisms underlying certain aspects remain at the stage of speculation. Additionally, some underlying mechanisms of the effect on Cesarean section on the infant have not been explored in depth. Therefore, future research could combine animal experiments, brain imaging, electroencephalography, and additional methods to further explore the neural mechanisms.

## Deficiencies of Existing Research and Prospects for Future Research

Previous studies have assessed the adverse effects of Cesarean birth through experimental designs, population cohort designs, clinical observations, and questionnaire investigations. Such studied attempted to reveal the mechanisms underlying the effect of Cesarean section through animal experiments and functional magnetic resonance imaging (fMRI). However, our review reveals deficiencies in the extant literature. Based on our review, we propose the following topics for future research.

In terms of methodology, most previous findings utilized cross-sectional designs, such as prospective cohort studies and crowd cohort studies, but few were based on longitudinal tracking studies. The influence of Cesarean delivery on children’s psychology is not static but may be different at different stages of development and periods of growth. Therefore, conclusions based on cross-sectional research lack continuity and generality. It would be appropriate to trace development to the primary school stage, to detect whether a dynamic process of change occurs, and over what time-scale and thereby obtain more robust conclusions. Additionally, researchers have recently proposed a more effective research method, named the sibling-control design, which compares Cesarean birth and spontaneous birth from the same parent. This design can largely balance the influence of the genetic and family environment, and thus improve the reliability of the research (Curran et al., 2015a). However, the drawback of this design is that the available sample is very limited.

Extant research topics are limited in scope and have not addressed some aspects of children’s psychology. The fetus delivered via the birth canal experiences a series of extrusions, efforts, and movements (flexion, internal rotation, extension, and so on), which continue through various planes of the birth canal until the delivery is complete. This early external pressure and the serial efforts of the fetus in the birth canal may have an impact on the child’s later persistence and willpower. A child who was delivered by Cesarean section did not experience this process, and its persistence and willpower may be negatively affected. Therefore, future research should explore whether Cesarean delivery indeed diminishes children’s persistence and willpower.

In terms of research participants, several previous studies compared two groups of subjects only: a Cesarean section group and a natural delivery group. However, a Cesarean birth may be due to medical or social factors, which may have different effects on the child’s psychology. If Cesarean births are not divided into these two groups, any conclusion is suspect. In addition, Cesarean births due to social factors can be classified as full-term Cesarean s section or premature Cesarean sections, which may also have different effects on the child. Therefore, future research should refine the choice of subjects.

Previous findings indicate the importance of the effects of general anesthesia in Cesarean births. General anesthesia increases the risk of autism in children, and girls born by Cesarean section are twice as likely to develop autism as boys (Chien et al., 2015). Therefore, future studies are needed to explore the effects of general anesthesia and its mechanisms on infants’ early development, and to further identify the intrinsic mechanisms underlying autism in men and women.

Vitale et al. (2016) noted that some studies report an increased risk of delayed motor and neurological development, generalized cognitive deficits, and learning difficulties in children born from mothers with psychosis. Therefore, it is necessary to comprehensively consider diverse factors when studying the relationship between Cesarean section and children’s neuropsychiatric development.

In terms of general orientation, attention should be paid to cross-cultural research. Previous studies have shown that different cultures have different attitudes toward Cesarean section (Savage, 1986). In some Asian cultures, Cesarean section is regarded as highly negative thing (Savage, 1986). However, in Brazil, Cesarean section is regarded as a symbol of modernization and is seen as positive and valuable (Nuttall, 2000). Therefore, it is necessary to consider cultural factors and role models when studying the influence of Cesarean section on children’s psychology. Researchers should compare births among different cultural groups to avoid drawing erroneous conclusions.

In terms of the clinical application of research, it is necessary to develop research into countermeasures. Accordingly, medical, psychological, and sociological researchers should form multidisciplinary research teams to enhance interdisciplinary cooperation, and thereby present effective measures to avoid unnecessary Cesarean sections.

## Conclusion

To conclude, Cesarean section may have adverse effects on children’s perceptual and sensory integration abilities and on the mother-child relationship, while the effects of Cesarean section on ADHD and ASD in children need to be further explored. However, the negative effects of Cesarean section have attracted insufficient attention in society at large. It is therefore important to improve the quality of information on these effects and disseminate it as widely as possible to improve children’s health.

## Author Contributions

HC gathered and analyzed the literature, and wrote and revised the manuscript. DT put forward the research topic and revised the manuscript.

## Conflict of Interest Statement

The authors declare that the research was conducted in the absence of any commercial or financial relationships that could be construed as a potential conflict of interest.
